# Uncontrolled Blood Pressure and Associated Factors Among Persons With Diabetes: A Community Based Study From Kerala, India

**DOI:** 10.3389/fpubh.2021.778235

**Published:** 2022-02-03

**Authors:** Aswathy Sreedevi, Vijayakumar Krishnapillai, Vishnu B. Menon, Minu Maria Mathew, Rajeesh R. Nair, Gopal S. Pillai, Mathews Numpelil, Jaideep Menon, Vishal Marwaha

**Affiliations:** ^1^Amrita Institute of Medical Sciences, Amrita Vishwa Vidyapeetham, Kochi, India; ^2^District Program Manager, National Health Mission, Ernakulam, India

**Keywords:** type 2 diabetes mellitus, blood pressure, diabetes complications, coexistent disease, diabetic retinopathy, peripheral arterial disease, diabetic neuropathies

## Abstract

The coexistence of raised blood pressure (BP) in people with type 2 diabetes mellitus (T2DM) is a major contributor to the development and progression of both macrovascular and microvascular complications. The aim of our study was to determine the prevalence of uncontrolled BP and its associated factors in persons with T2DM in a district in Kerala.

**Methods:**

The study was conducted in Ernakulam district in Kerala, and a total of 3,092 individuals with T2DM were enrolled after obtaining consent. Those with a BP “above or equal to 140 mmHg” and/or “above or equal to 90 mmHg” were thus considered to have uncontrolled BP. If the BP was equal or >140 and/or 90 mmHg, a repeat reading was taken after 30 min and the average of the two was considered. Basic demographic details were enquired along with electronic measurement of BP, HbA1c estimation and screening for diabetic retinopathy, peripheral arterial disease (PAD), and peripheral neuropathy. Quantitative and qualitative variables were expressed as mean (SD) and proportions, respectively. The model for determinants of uncontrolled BP was developed adjusting for age, gender, education, duration of diabetes, occupation, body mass index (BMI) and clustering effect.

**Results:**

The mean age of the study population was 59.51 ± 9.84 years. The mean duration of T2DM was found to be 11.3 ± 6.64 years. The proportion of uncontrolled HTN adjusted for clustering was 60% (95% CI 58 and 62%). Among them, only one in two persons (53.3%) had a history of hypertension. Age >60 years [adjusted odds ratio (aOR) 1.48, 95% CI 1.24, 1.76; *p* < 0.001], unemployment (aOR 1.33, 95% CI 1.01, 1.75; *p* < 0.01), duration of diabetes > 11 years (aOR 1.42, 95% CI 1.19, 1.68; *p* < 0.001), and BMI ≥23 (aOR 1.33, 95% CI 1.10, 1.59; *p* < 0.002) were found to be independent determinants of high BP levels when adjusted for the aforementioned variables, gender, education, and cluster effect. The association between complications, such as peripheral neuropathy, PAD, and retinopathy showed a higher risk among those with uncontrolled BP. Retinopathy was 1.35 times more (95% CI 1.02, 1.7, *p* < 0.03), PAD was 1.6 times more (95% CI 1.2, 2.07, *p* < 0.001), and peripheral neuropathy was 1.5 (95% CI 1.14, 1.9, *p* < 0.003) times more compared to their counterparts.

**Conclusion:**

Target BP levels were far from being achieved in a good majority of the persons with T2DM. To reduce further macrovascular and microvascular events among people with T2DM, effective awareness and more stringent screening measures need to be employed in this population.

## Introduction

Type 2 diabetes mellitus (DM) is a metabolic disorder characterized by insulin resistance and insulin hyposecretion that result in hyperglycaemia. It is estimated that by the year 2030, about 439 million adults (7.7%) will be affected by diabetes, globally (1). According to the Indian Council of Medical Research-India Diabetes (ICMR–INDIAB) study, the overall prevalence of diabetes in India is 7.3% (95% CI 7.0–7.5) (2), which is in tandem with the global estimates. Long-standing diabetes can pave the way for various microvascular and macrovascular complications, dementia, certain cancers, and respiratory disease (3, 4).

Hypertension is defined as a condition where the blood vessels have persistently raised pressure. The coexistence of hypertension or blood pressure (BP) above the target level in patients with type 2 diabetes mellitus (T2DM) is a major contributor to the development and progression of macrovascular and microvascular complications (5). The combined effect of these can seriously affect the health status of the population. Studies have shown that people with diabetes face a 2- to 4-fold increased risk of cardiovascular disease (CVD) when compared to the general population (6). In people with diabetes, coexisting hypertension can triple the risk of coronary artery disease (CAD), double the total mortality and stroke risk, and can be responsible for up to 75% of all CVD events (7). Hypertension has also been shown to accelerate the progression of certain complications, such as diabetic nephropathy, retinopathy, and neuropathy (8–10).

Bringing down BP has proven to be beneficial in reducing complications associated with diabetes. Several studies have shown that treating hypertension in people with diabetes can reduce atherosclerotic cardiovascular disease (ASCVD) events, heart failure, and microvascular complications (11). As per the Joint National Committee (JNC) eight guidelines, among people with diabetes, anti-hypertensive therapy should be initiated when BP is ≥140/90 mmHg, and the target BP should be maintained below 140/90 mmHg (12). In the UK Prospective Diabetes Study (UKPDS), compared to individuals in the control group, participants in the tight BP control group had a reduction of 34% and 37% risk of macrovascular diseases and microvascular disease, respectively (13).

However, maintaining the target range of BP is still a challenge. In a European study in 24 countries, a target BP level of <140/90 mmHg was achieved only in 54% of people with diabetes (14). In India, hypertension is still a major public health issue. Although there are significant regional differences, it is estimated that there are more than 200 million hypertensive individuals in the country (15). Studies have shown that among people with diabetes, hypertension often remains undiagnosed (16), thereby delaying therapy. Additional attention to traditional cardiovascular (CV) risk factors, such as high BP, could yield further substantive reductions in CV events and mortality in adults with diabetes (17). Data on hypertension control status among type 2 Diabetes in India are limited. The objectives of this study were to determine the prevalence of uncontrolled BP among persons with diabetes and to assess the associated factors, including.

## Methods

A community-based cross-sectional study was carried out in Ernakulam district in Kerala, India. The district, which is an administrative division in the state, has the highest population density and is the commercial capital of the state. A prevalence of 20.6% (16) was used to calculate the sample size from a previous study on hypertension/uncontrolled BP among persons with diabetes. With a relative precision of 10%, the sample size was calculated to be 1,425. As clusters were taken, a design effect of two was used to arrive at a sample size of 2,850.

A two-stage cluster sampling with population proportionate to size sampling (PPS) was carried out. In the first stage, 33 clusters, which are local self-government areas, were drawn by probability proportional to their size. The population of all the local self-government (LSG) areas was listed. The cumulative population was calculated. The total population was divided by the number of clusters to determine the sampling interval. The first number was picked by the random number table within the sampling interval. The corresponding LSG was selected. The sampling interval was added 33 times to get the 33 LSG areas, which are the clusters. The frontline health worker [accredited social health activist (ASHA)] of each ward provided the list of persons with diabetes to the Primary health centre (PHC). The team at the PPHC chose every third/fourth person from the list. Thus, about 110 persons were provided a referral card and referred considering a non-response rate of 20%.

The first 85–90 participants who came to the camp with diabetes for more than a year were enrolled in the study after obtaining informed consent. Local camps were conducted in the selected LSG areas under the aegis of an international non-government organization (NGO), a tertiary care center, Primary Health Center, and National Health Mission. Thus, a total of 3,092 persons with diabetes were enrolled. The inclusion criteria of the study included adults with type 2 diabetes of at least 1 year of duration. The exclusion criteria were those who could not respond to the questions with coherence or those who were cognitively impaired, pregnant woman, and above 80 years. However, in order to efficiently utilize resources, the screening for complications, such as retinopathy, peripheral arterial disease (PAD), and peripheral neuropathy, was carried out among persons with more than 5 years of diabetes. About 33 camps were conducted from November 2020 to March 2021 by a multidisciplinary team of community physicians, ophthalmologists, doctors with training in Podiatry, nurses, laboratory technician, optometrists, and medical social workers. Institutional ethical committee approval was obtained vide IEC-AIMS-2020-COMM-186 dated November 9, 2020.

The outcome variable was uncontrolled BP among persons with type 2 diabetes. The BP was considered to be controlled if the systolic and diastolic values were <140 and <90 mmHg. This was also synonymous with having attained target BP. Those with a BP “above or equal to 140 mmHg” and or “above or equal to 90 mmHg” were thus considered to have uncontrolled BP. The BP was measured by the OMRON HEM 7124 automatic blood pressure monitor (Shimogyo-ku, Kyoto, Japan) by measuring upper arm BP. If a level above or equal to 140 and or 90 mmHg was observed, the measurement was repeated after 30 min and the average of the two readings was taken (18). Several guidelines have prescribed a BP target of not more than >140 and >90 mmHg (11) for persons with diabetes. The independent variables collected included sociodemographic details, anthropometric measurements, such as weight and height using standard measurements, self-reported co-morbidity, personal habits, such as tobacco and alcohol, known complications of diabetes, duration of illness, family history of diabetes, and Glycosylated Haemoglobin (HbA1c). HbA1c was measured with a point-of-care device HbA1c HemoCue auto analyzer after validation with the laboratory values. A correlation of 0.9 was obtained with the laboratory values. The targets for glycated hemoglobin were as follows: <7% as ideal, ≥7 to <8 satisfactory, and ≥8 unsatisfactory (19). Assessment of foot complications, such as PAD, peripheral neuropathy in the lower limbs, and retinopathy, was also carried out. Body mass index (BMI) was calculated from the weight in kilogram (kg) and height in metre (m) measurement, and the Asian standards were used for categorization; 18.5–22.9 for normal, 23–27.5 for overweight, and >27.5 for obese.

After lying down and being made comfortable, the vibration perception threshold (VPT) was tested using a biothesiometer. A probe was placed in the palm of patients to familiarize them with the vibration perception. The patient was advised to feel the vibration on his/her feet and slowly vibration intensity was increased. At the point at which the patient felt the vibration, the VPT was recorded in volts and graded. This indicated the threshold voltage that can be perceived by the person. The probe was applied to the big toe and medial malleoli. The vibration intensity was increased gradually by turning the dial. The VPT value was graded as <15 volts as normal (Grade I), 16–20 volts as mild loss of sensation (Grade II), 21–25 volts as moderate loss of sensation (Grade III), and >25 volts as severe and abnormal (Grade IV) (11).

The patient continued to be in the lying posture, and ankle-brachial pressure index was measured to detect PAD. First brachial BP was measured using a sphygmomanometer and handheld Doppler, then the ankle pressure of each leg was measured, and the ratio of ankle pressure to brachial pressure was calculated for the left and right lower limbs. The BP cuff was placed on the arm, with the limb at the level of the heart. The ultrasound gel was applied in the antecubital fossa over the patient's brachial pulse. The transducer of the handheld Doppler was placed over the antecubital fossa on the gel, and the transducer was positioned to maximize the intensity of the signal. The cuff was then inflated to about 10 mmHg above the expected systolic BP of the patient such that, the Doppler signal disappeared. The cuff was then deflated at approximately 1 mmHg/s. When the Doppler signal re-appears, the pressure of the cuff is recorded as brachial systolic pressure. To measure ankle pressure, the cuff was placed immediately proximal to the malleoli. The ultrasound gel was applied on the skin overlying the dorsalis pedis (DP) artery in the foot. The Doppler signal of the DP artery was found slightly lateral to the midline of the dorsum of the foot. Using a standard handheld Doppler probe and the ultrasound gel, the signal was located. The cuff was inflated till the Doppler signal was no longer heard. Then using the same technique, the cuff was deflated until the Doppler signal re-appeared. The measurement was recorded. The Ankle Brachial Index (ABI) was calculated for each leg. The ABI value was determined by taking the higher pressure of the two arteries at the ankle, divided by the brachial arterial systolic pressure. In calculating the ABI, the higher of the two brachial systolic pressure measurements was used. In normal individuals, there should be a minimal (<10 mmHg) interarm systolic pressure gradient during a routine examination. A reading ≥1.3 was considered to be abnormal vessel hardening, 0.9–1.2 to be normal, 0.50–0.79 to be moderate arterial disease, under 0.50 considered as severe arterial disease (11).

Retinopathy was assessed by mydriatic fundus photography and rechecked by indirect ophthalmoscopy. All patients underwent visual acuity examination with available glass correction and pinhole to see if there was any improvement with a further change of glasses. All patients were dilated with tropicamide eye drops and mydriatic retinal photography was performed. All patients also underwent retinal examination with an indirect ophthalmoscopy by a trained ophthalmologist and retinal findings and diagnosis were confirmed. Grading of diabetic retinopathy was done on site and confirmed with viewing the retinal photographs by experts.

The data collected were entered in excel and data analysis was carried out in Statistical Package of Social Sciences (SPSS) (20, 24). For the purposes of this study, multiple morbidities were defined as the presence of more than one morbidity in a person with diabetes, such as heart disease, thyroid disease, and hyperlipidaemia. The quantitative variables have been expressed as mean and SD and the qualitative as proportions. The bivariate analysis was done by the chi-square test. The proportion of uncontrolled HTN adjusted for clustering has been reported. Multiple variable analysis adjusted for clustering (number of camps) along with variables that showed *p* < 0.1 in the univariate analysis was carried out. Age, gender, duration of DM, education, BMI, and occupation were considered as fixed effects, and cluster was considered as random effect in the logistic regression model. Adjusted odds ratio and 95% CI are reported. This was carried out in STATA 15 (College Station, TX, USA).

## Results

The mean age of the study population was 59.51 years ± 9.84, and it ranged from 29 to 80 years. There was an almost equal distribution among persons less than or equal to 60 years, 1,423 (46.2%) and above 1,652 (53.7%). Men constituted only about a third [1,144 (37%)] of the participants and more than three-quarters [2.422 (78.4%)] of the respondents were from rural areas (Table 1). However, all study participants were literate and only 111 (6.7%) had more than 12 years of schooling. About half [1,478 (49.5%)] were below the poverty line according to self-reports. The mean duration of diabetes was 11.2 ± 6.64 years. Only 10.8% had an ideal HbA1c below 7. As far as the cardiometabolic risk factors were concerned, only about a quarter [821 (27%)] had a BMI of <23 as per the ideal Asian standards. More than a half (60.1%) had BP equal to or above 140/90 mmHg of which more than a half (966/1,812) (53.3%) were known hypertensives. The proportion of uncontrolled Hypertension (HTN) adjusted for clustering was 60% (95% CI 58 and 62%).

**Table 1:** Sociodemographic distribution of the study population.

	**Frequency**	**Percentage**
**Age (in years)**		
≤ 60	1,423	46.3%
>60	1,652	53.7%
**Gender**		
Men	1,144	37%
Women	1,948	63%
**Place of residence**		
Rural	2,422	78.3%
Urban	670	21.7%
**Education**		
≤ 12 years of schooling	2,618	92.6%
>12 years of schooling	210	6.8%
*264 missing		
**Socioeconomic status**		
Non priority group	1,505	50.5
Priority group	1,478	49.5
*109 missing		
**Occupation**		
Unemployed	619	21
Home maker	1,119	38
Employed / Retired	1,206	41
*148 missing		
	**Frequency**	**Percentage**
**HbA1C** **(Glycosylated hemoglobin)**		
Ideal	302	10.8
Satisfactory	522	18.7
Unsatisfactory	1,968	70.5
**Duration of diabetes (in years)**		
≤ 11	1,865	60.3
>11	1,193	38.6
*34 missing		
**Body Mass Index (BMI)**		
<23	821	27
≥23	2,216	71.7
**Blood pressure controlled**		
Yes	1,205	39.9
No	1,812	60.1
**Known hypertension among those**		
**with uncontrolled blood pressure**		
Yes	966	53.3
No	846	46.7
**Peripheral neuropathy**		
Yes	988	53.8
No	847	46.2
**Peripheral arterial disease**		
Yes	738	48.5
No	783	51.5
**Retinopathy**		
Yes	612	28.9
No	1,501	71.1

Thus, the target BP for persons with diabetes was achieved by only 1,205 (39.9%) patients. Complications, such as PAD and peripheral neuropathy, were found among about a half [738 (48.5%)] and more than a half [963 (53.5%)], respectively. Retinopathy was found among more than a fourth, i.e., 612 (28.9%).

The BP target level was not attained among 65.5% of those aged more than 60 years compared to 53.5% among those who were <60 years (*p* < 0.001). Women had a significantly higher percentage of uncontrolled BP at 61.7% (*p* < 0.019). Uncontrolled BP was higher among those with a duration of diabetes of more than 11 years (*p* < 0.001). The uncontrolled BP was found to significantly decrease with the improvement of employment status from 66.2 to 54.6% (*p* < 0.001). BP was significantly above the target level in those with a BMI ≥23. Others, such as rural-urban residence, education, socioeconomic status, physical activity, Hba1c, heart disease, and respiratory disease, were not found to be significant (Table 1).

The multiple logistic regression was used by the enter method, and the following variables were found to be independent predictors. Age >60 years [adjusted odds ratio (aOR) 1.48, 95% CI 1.24, 1.76; *p* < 0.001], unemployment (aOR 1.33, 95% CI 1.01, 1.75; *p* < 0.01), duration of diabetes (aOR 1.42, 95% CI 1.19, 1.68; *p* < 0.001), BMI ≤ 23 (aOR 1.33, 95% CI 1.10, 1.59; *p* < 0.002) were found to be independent predictors of high BP levels when adjusted for the aforementioned variables, education, gender, and cluster (Table 2). For the final multivariate analysis, 2,588 samples were considered. However, there was a loss of 16% of samples in the analysis for covariates, the reverse calculation of the power for each significant variable was 95%, which is sufficient to establish risk.

**Table 2:** Factors associated with uncontrolled blood pressure adjusted for clustering.

	**Controlled**	**Uncontrolled**	**Unadjusted OR (95% CI)**	**Adjusted OR (95% CI)**	***p***
**Age(in years)**					
≤ 60	646 (46.5)	742 (53.5)	1	1	
>60	556 (34.5)	1,056 (65.5)	1.65 (1.42, 1.92)	1.48 (1.24, 1.76)	<0.001
**Sex**					
Male	476 (43.7)	639 (57.3)	1	1	
Female	729 (38.3)	1,173 (61.7)	1.19 (1.03, 1.39)	1.06 (0.84, 1.34)	0.373
**Duration of DM**					
< =11.2 yrs	792 (43.5)	1,027 (56.5)	1	1	
>11.2 yrs	400 (34.3)	766 (65.7)	1.47 (1.26, 1.72)	1.42 (1.19, 1.68)	<0.001
**Education**					
< =12 yrs	1,021 (39.8)	1,541 (60.2)	1	1	
>12 yrs	96 (46.4)	111 (53.6)	0.76 (0.57, 1.02)	0.87 (0.67, 1.12)	0.21
**Body mass index**					
<23	354 (44.0)	450 (56.0)	1	1	
≥23	838 (38.5)	1,340 (61.5)	1.25 (1.07, 1.48)	1.33 (1.10, 1.59)	<0.002
**Occupation**					
Employed	536 (45.4)	644 (54.6)	1	1	
Unemployed	204 (33.8)	399 (66.2)	1.62 (1.33, 1.99)	1.33 (1.01, 1.75)	0.005
Home maker	417 (38.1)	678 (61.9)	1.35 (1.14, 1.59)	1.22 (0.92, 1.63)	0.112

The association between complications, such as peripheral neuropathy, PAD, and retinopathy, showed a higher risk among those with uncontrolled BP. Retinopathy was 1.35 times more (95% CI 1.02, 1.7, *p* < 0.03), PAD was 1.6 times more (95% CI 1.2, 2.07, *p* < 0.001), and peripheral neuropathy was 1.5 (95% CI 1.14, 1.9, *p* < 0.003) times more (Table 3) compared to their counterparts.

**Table 3:** Association between uncontrolled blood pressure among persons with type 2 diabetes and complications.

**Uncontrolled blood pressure**						
	**Yes**	**No**	**Total**	**COR(95% CI)**	**aOR(95% CI)**	**p**
**Retinopathy**						
Yes	401 (65.5)	211 (34.5)	612	1.45 (1.19, 1.77)	1.35 (1.02, 1.77)	0.03
No	850 (56.6)	651 (43.4)	1,501	1	1	
**Peripheral arterial disease**						
Yes	503 (68.1)	235 (31.9)	738	1.43 (1.16, 1.76)	1.6 (1.23, 2.07)	0.00
No	469 (59.8)	314 (40.2)	783	1		
**Peripheral neuropathy**						
Yes	635 (65.8)	328 (34.2)	965	1.37 (1.19, 1.77)	1.48 (1.14, 1.92)	0.003
No	489 (66.3)	348 (33.7)	737	1		

## Discussion

Six out of 10 persons with type 2 diabetes in our study had BP above the target level. Age above 60 years, duration of diabetes of more than 11 years, a BMI above or equal to 23, and unemployment were independent determinants of high BP.

There are not many studies in India, which have looked at the control of BP among persons with diabetes. Of a few, some have looked at the coexistence of hypertension and diabetes which was 20% (16), and another hospital-based study has found uncontrolled BP to be high at 70% (21). Global studies in Europe and USA also report uncontrolled BP proportion ranging from 54 (14) to 68.4% (22), respectively, whereas it was only about a third i.e., (34%), in Spain (23). This calls for more attention to control BP particularly among persons with diabetes as there is 1.5–2 times increased occurrence of hypertension among persons with diabetes in India and this coexistence has seen an increase (24). It is also of concern that among those with uncontrolled BP, only a half i.e., (53.3%), were known hypertensives. The complications, such as PAD, diabetic retinopathy, and diabetic neuropathy, have also been found to be significantly higher among those with uncontrolled BP in this study. However, as it is a cross-sectional study, the temporality cannot be determined, as to whether the high BP led to complications or the complications led to higher blood pressure.

The overall prevalence of diabetic retinopathy was 28.7%. Although this is slightly less than the global prevalence of diabetic retinopathy 34.6% reported by Yau et al. (25), a similar prevalence was reported a decade earlier in a smaller population of self-reported diabetics (26). Diabetic retinopathy is one of the leading causes of blindness among persons of working age (27) and hypertension plays a critical role in the occurrence and progression of the microvascular complications, such as diabetic retinopathy and neuropathy (5). The UKPDS study had shown a 34% reduction in the rate of progression of diabetic retinopathy when the BP was kept below the target value of <150/85 mmHg (13). Proper screening and management of hypertension among people with diabetes will help to reduce the burden in the longer run. Around 54% (988) of the individuals had peripheral neuropathy. Several studies in the past had shown a higher prevalence of diabetic neuropathy (28–30). The International Prevalence and Treatment of Diabetes and Depression Study (INTERPRET-DD) (31) that collected data from 14 countries had shown an overall prevalence of 26.7%, though, it was 13.3% in India. However, this may not be representative of India/Kerala as a sample of only 188 were studied and the area of study is not mentioned. Prevalence of PAD was also on the higher side, with about 48% of people diagnosed with the same in our study. Global estimates of PAD (32) showed a reduced prevalence in low- and middle-income countries, with a majority of them living in southeast Asian region. Both DM and hypertension have been found to be significantly associated with PAD (33). Thus, reducing complications, such as PAD, can be effectively achieved by reaching target BPs in persons with diabetes.

Thus, there is an urgent need to screen the BP of persons with diabetes. However, the metabolic control for persons with diabetes is a BP <140/90 mmHg (19), a large percentage of this diabetic population seem far from achieving it.

Three-quarters of the study population had a BMI ≥23. People with diabetes who had a BMI of more than or equal to 23 were found to have their BP values above the target level. Obesity has long been associated with hypertension (34, 35) and is a major contributor to morbidity and mortality among people with diabetes. Recommendations from the various associations, namely, the ICMR (19), the American Diabetes Association, and the European Association for the Study of Diabetes, have emphasized the management of obesity and hypertension to reduce CV events among people with diabetes (36). Therefore, identifying people with diabetes who are currently leading a sedentary lifestyle is of utmost importance and measures need to be taken to increase physical activity in such individuals.

Individuals aged above 60 years and increasing duration of diabetes were also independent determinants of uncontrolled BP. Age is known to be a major predisposing factor for most of the common degenerative conditions. The risk of hypertension in the general population can double with every 9–10-year age increment (37).

## Limitations

There may be problems with generalizability as people belonging to low- and middle socioeconomic status are more likely to attend these camps than those of high socioeconomic status. As it was a camp, setting the BP could not be measured two times for everybody and could only be measured for those who had a reading ≥140/90 mmHg. This study was conducted during the lull after the first wave of the Coronavirus Disease-2019 (COVID) pandemic and before the second wave started in Kerala. Therefore, it is difficult to ascertain whether COVID may have contributed to higher BP levels.

## Conclusion

The target levels of BP among people with type 2 diabetes are far from being achieved. This needs emphasis through patient and physician awareness. Increased BP has been associated with micro- and macro-vascular complications, such as retinopathy, neuropathy, and PAD, respectively. Control of BP to below target levels is thus very important for persons with diabetes.

## Data Availability Statement

The datasets presented in this article are not readily available because permissions will be required from Amrita Institute of Medical Sciences (Kochi), National Health Mission (Ernakulam) and Lions Clubs International (District 318C) for release of data. Requests to access the datasets should be directed to Amrita Institute of Medical Sciences, Kochi.

## Ethics Statement

The studies involving human participants were reviewed and approved by IEC Amrita Institute of Medical Sciences, Kochi. The patients/participants provided their written informed consent to participate in this study.

## Author Contributions

AS contributed to study conception, data collection, interpretation of data, and drafting the manuscript. VK contributed to conception, implementation, and editing of manuscript. VMe contributed to conduct, data collection, and editing of manuscript. MM analysed the data. RN contributed to conduct, data collection, and editing of the manuscript. GP contributed to conception, implementation, and data collection. MN contributed to study conception and supported in data collection. JM contributed to conception and editing of the manuscript. VMa contributed to conduct of the study and editing of the manuscript. All authors read and approved the manuscript and critically revised the manuscript for important intellectual content.

## Funding

This work was funded from Lion Club International Foundation (DIA16895/318-C).

## Conflict of Interest

The authors declare that the research was conducted in the absence of any commercial or financial relationships that could be construed as a potential conflict of interest.

## Publisher's Note

All claims expressed in this article are solely those of the authors and do not necessarily represent those of their affiliated organizations, or those of the publisher, the editors and the reviewers. Any product that may be evaluated in this article, or claim that may be made by its manufacturer, is not guaranteed or endorsed by the publisher.
